# Personalized Tacrolimus Dose Requirement by CYP3A5 but Not ABCB1 or ACE Genotyping in Both Recipient and Donor after Pediatric Liver Transplantation

**DOI:** 10.1371/journal.pone.0109464

**Published:** 2014-10-13

**Authors:** Yi-kuan Chen, Long-zhi Han, Feng Xue, Cong-huan Shen, Jun Lu, Tai-hua Yang, Jian-jun Zhang, Qiang Xia

**Affiliations:** Department of Liver Surgery and Liver Transplantation, Ren Ji Hospital, School of Medicine, Shanghai Jiao Tong University, Shanghai, P.R. China; Centre for Inflammation Research, United Kingdom

## Abstract

Tacrolimus (TAC) is the backbone of an immunosuppressive drug used in most solid organ transplant recipients. A single nucleotide polymorphism (SNP) at position 6986G>A in *CYP3A5* has been notably involved in the pharmacokinetic variability of TAC. It is hypothesized that *CYP3A5* genotyping in patients may provide a guideline for TAC therapeutic regimen. To further evaluate the impact of *CYP3A5* variants in donors and recipients, *ABCB1* and *ACE* SNPs in recipients on TAC disposition, clinical and laboratory data were retrospectively reviewed from 90 pediatric patients with liver transplantation and their corresponding donors after 1 year of transplantation. The recipients with *CYP3A5* *1/*1 or *1/*3 required more time to achieve TAC therapeutic range during the induction phase, and needed more upward dose during the late induction and the maintained phases, with lower C/D ratio, compared with those with *CYP3A5* *3/*3. And donor *CYP3A5* genotypes were found to impact on TAC trough concentrations after liver transplantation. No association between *ABCB1* or *ACE* genotypes and TAC disposition post-transplantation was found. These results strongly suggest that *CYP3A5* genotyping both in recipient and donor, not *ABCB1* or *ACE* is necessary for establishing a personalized TAC dosage regimen in pediatric liver transplant patients.

## Introduction

Tacrolimus (TAC) is the backbone of immunosuppressive drug used worldwide in organ transplantation and characterized by a narrow therapeutic range and high inter-individual variability in its pharmacokinetics [1],[2]. To achieve the desired target blood concentrations is of critical importance to avoid rejection and dose-related adverse effects after transplantation [3]. The variability makes it difficult to establish an empirical dose regimen for this drug, especially in pediatric patients, in whom 100-fold variability in pharmacokinetic parameters and blood concentration after a fixed dose is routinely observed [4], [5]. Underexposure to TAC may result in immunosuppression failure and acute rejection in recipients. On the other hand, overexposure to it may put patients at risk for its considerable toxicity. Therefore, maintaining the drug exposure within this narrow safe therapeutic window becomes a critical aspect in patient management. Concerning the concept that young children need a higher TAC dose than adult patients [4], [6], the blood TAC concentration should be monitored regularly to maintain a therapeutic range, especially during the induction phase post-transplantation therapy, when the risk of rejection is the highest. Although various factors, such as age, sex, body weight, drug interactions and other factors lead to the wide range of interpatient variability ineffective dosage of TAC [7], among them genetic factors play a critical role in the pharmacokinetic properties and therapeutic levels of TAC.

Cytochrome P450 (CYP) 3A5 is the major enzyme responsible for the metabolism of TAC and is found in small intestine as well as in the liver [8]. A single nucleotide polymorphism (SNP) in the *CYP3A5* gene involving an A to G transition at position 6986 within intron 3 was found strongly associated with CYP3A5 protein expression. At least one CYP3A5*1 allele were found to express large amounts of CYP3A5 protein, whereas homozygous for the CYP3A5*3 allele did not express significant quantities of CYP3A5 protein, which causes alternative splicing and results in a truncated protein and a severe decrease of functional *CYP3A5* [9]. It has become clear that CYP3A5*1/*1 or *1/*3 (hereinafter defined ‘expressor’) are significantly associated with lower dose-adjusted TAC exposure and increased TAC dose requirements in order to achieve target blood concentrations compared with variant CYP3A5*3/*3 (hereinafter defined ‘nonexpressor’) [7], . However, it is controversial that, for liver transplantation, the impact of the *CYP3A5* genotype of both the recipients (intestine) and the donors (graft liver) should be taken into account when evaluating TAC pharmacokinetics.

TAC is also substrate of P-glycoprotein, a member drug efflux transporter encoded by the multidrug resistance *ABCB1* gene [13], [14]. It has been suggested that some SNPs of the *ABCB1* gene in exons 12 (1236C>T), 21 (2677G>A/T) and 26 (3435C>T) maybe affect synthesis and function of P-glycoprotein. In addition, angiotensin converting enzyme (ACE), which is a key enzyme in the renin-angiotensin system, catalyzes the conversion of angiotensin I to II in the liver and kidney. A line of evidence suggests that variation in intron 16 of the ACE gene (14091–14378) may impact on pharmacokinetics and pharmacodynamics of TAC [15]. However, the impact of SNPs of *ABCB1* and *ACE* on pediatric liver transplants remains unclear.

Although much effort has been devoted to the better understanding of inter-individual differences in response to TAC, little data are available about these relationships in Chinese liver transplanted recipients [16], [17], particularly in the pediatric population. Moreover, the effects of *CYP3A5, ABCB1* and *ACE* variants on clinical outcomes are not well established in China. The aim of this study was, therefore to retrospectively determine the impact of *CYP3A5* genotype of recipients (intestine) and donors (graft liver), age, sex, body weight, primary diseases and other factors on TAC dosing requirements and disposition in a cohort of pediatric liver recipients during the 12 months following transplantation. We evaluated the effect of *CYP3A5, ABCB1* and *ACE* variants on the clinical outcomes in our pediatric liver recipients, and attempted understanding the relationship between *CYP3A5*, *ABCB1* or *ACE* genotype and TAC pharmacokinetics may improve our knowledge of how to most effectively administer this drug, leading to considerable benefit to pediatric liver transplant patients.

## Materials and Methods

### 1. Patients

The patients in this retrospective study were 90 consecutive de novo liver graft recipients who underwent living-donor liver transplantation at Shanghai Ren Ji Hospital between October 2008 and December 2012. Median age of the pediatric patients at liver transplantation was 10 months (range, 5–72 months). This study was reviewed and approved by Shanghai Jiao Tong University School of Medicine Ren Ji Hospital Ethical Board (Approval No.: 2013010), and written informed consent was obtained from all their parents during enrollment.

All pediatric patients were administered the immunosuppressive therapy on day 2 to 3 after liver transplantation. TAC (Astella Pharma Co., Limited) was administered orally (dissolved in water for young children) twice daily with an initial dose of 0.15 mg/kg/day, and subsequently adjusted to archiving target blood trough concentration (termed C_0_) through routine monitoring. The target C_0_ was between 10 and 15 ng/ml during the first month, between 8 and 12 ng/ml during 2–6 months, and between 5 and 8 ng/ml thereafter. In general, repeat or multiple post-operative infections were considered as over-immunosupressive, which needs to reduce the dose of TAC, whereas when an acute cellular rejection happens, it was considered as under-immunosupressive, which needs to increase the dose of TAC. For acute cellular rejection cases, additional immunosuppressive therapy consisted of a maintenance dose of mycophenolate mofitil and steroid.

### 2. Tacrolimus C_0_ monitoring and C/D ratio assessment

Analysis of all patients’ clinical and laboratory assessments on day 3, 7, 14, and in month 1, 3, 6, and 12 post-transplantation were performed. EDTA-treated blood (1 ml) was collected every 12 h after the previous dose and then blood TAC C_0_ was measured by a microparticulate enzyme immunoassay (Abbott Co., Ltd, Tokyo, Japan). The daily dose of TAC was recorded and weight-adjusted dosage (mg/kg/day) was calculated. The blood concentration was measured and normalized using the corresponding dose. A dose ratio was obtained by the concentration/dose (C/D) ratio, which was used for estimating TAC concentration. When the blood TAC C_0_ was not measured at a given time point, the data were excluded.

### 3. Genotyping of *CYP3A5, ABCB1* and *ACE*

The 90 pediatric recipients and 90 adult donors were genotyped for the single nucleotide polymorphism of *CYP3A5* at position 6986A>G (the *3 or *1 allele, rs776746), *ABCB1* at exons 12 (1236C>T, rs1128503), 21 (2677G>A/T, rs2032582) and 26 (3435C>T, rs1045642) and *ACE* at intron 16 (14091–14378). The genotyping was detected using the PCR-based sequencing. In brief, whole blood samples (1.0 ml) were collected in EDTA-treated tubes. The genomic DNAs were extracted from leukocytes with a QIAamp Blood kit (Qiagen, Hilden, Germany). A fragment containing the 6986A>G polymorphism was amplified in ABI 7900 system (Applied biosystems, Foster City, CA, USA), using Taq polymerase qPCR kit (TaKaRa Bio. Inc., Dalian, China). The primers 5′-ACTGCCCTTGCAGCATTTA-3′ (forward) and 5′-CCAGGAAGCCAGACTTTGA-3′ (reverse) for *CYP3A5*, primers 5′-ACTTCAGTTACCCATCTCG-3′ (forward) and 5′-TTTCCCGTAGAAACCTTAC-3′ (reverse) (1236C>T), primers 5′-ATAGCAAATCTTGGGACAG-3′ (forward) and 5′-GCATAGTAAGCAGTAGGGA-3′ (reverse) (2677G>A/T), primers 5′-TGGCAGTTTCAGTGTAAGA-3′ (forward) and 5′-CTCCCAGGCTGTTTATTTG-3′ (reverse) (3435C>T) for *ACBC1* and primers 5′-GCCCTGCAGGTGTCTGCAGCATGT-3′ (forward) and 5′-GGATGGCTCTCCCCGCCTTGTCTC-3′ (reverse) (1st), primers 5′-TGGGACCACAGCGCCCGCCACTAC-3′ (forward) and 5′-TCGCCAGCCCTCCCATGCCCATAA-3′ (reverse) (2nd) for *ACE* were employed. The qPCR process was carried out as following: 95°C for 10 min, then 94°C for 30 s, 55°C for 30 s, 72°C for 60 s for total 40 cycles and finally 72°C for 7 min. The products were then purified with a QIAquick PCRPurification kit (Qiagen, Hilden, Germany) and run on an ABI 3730XL Genetic Analyzer (Applied biosystems, Foster City, CA, USA) according to the manufacturer’s recommendations.

### 4. Outcome measures

The primary outcomes were TAC dosing requirement (normalized for body weight) and C/D ratio (the latter as surrogate marker) at indicated time points of day (d) 3, d 7, d 14, month (m) 1, m 3, m 6 and m12 for TAC clearance. Secondary outcome measures were acute rejection, acute and chronic infection, as well as liver function.

A one-year follow up after liver transplantation was performed to investigate the possible correlation of various infections with the CYP3A5*1 status of donors and recipients, and with the SNPs of *ABCB1* and *ACE* of recipients. Incidence of post-operative infections and acute cellular rejection were determined by double-blind physicians. Viral infections were classified by viral pathogens, including CMV, EBV, rotavirus, herpes virus, and HBV. The overlap and relative severity of these infections were also recorded. Viral infections differed according to the intensity of immunosuppression and the serologic status of the recipient. Diagnosis of acute cellular rejection or immunosuppressant-induced hepatic toxicity was based on pathological criteria.

### 5. Statistical analyses

All data were collected and expressed as the mean ± standard deviation or the median with deviation range. Data among several groups or continuous variables between two groups were compared using one-way ANOVA, while continuous variables among several groups were compared using two-way ANOVA analysis and followed by Bonferroni adjustment. Categorical variables were compared using Chi-square test or Fisher’s exact test. Other data between two groups was analyzed with T-test. A value *p*<0.05 was considered statistically significant in all analyses, which were performed using SPSS 19.0 soft (SPSS inc., Chicago, IL).

## Results

### 1. Pediatric patient clinical characteristics

We summarized the demographic characteristics of the patients and showed them in Table 1. The total of 90 eligible Chinese pediatric liver transplant recipients (52 boys and 38 girls) and 90 Chinese healthy donors (37 men and 53 women) were enrolled. The median age of patient age was 10 months (between 4 months and 10 years), whereas that of donor age median was 30 years (between 21 and 56 years). The primary diseases of the pediatric recipients included 89 congenital biliary atresias (98.9%) and 1 postoperative chologenic infection (1.1%). CYP3A5*1/*1 (AA allele), *CYP3A*5*1/*3 (AG allele) and CYP3A5*3/*3 (GG allele) were 3 (3.3%), 37 (41.1%) and 50 (55.6%) cases respectively in recipients, whereas three variants were 11 (12.2%), 34 (37.8%) and 45 (50%) cases in donors. The allele frequencies of *ABCB1* 1236CT, TT, CC, 2677AT, GA, GG, GT, TT, 3435CC, CT and TT were 42.2%, 46.7%, 11.1%, 14.4%, 12.2%, 23.4%, 36.7%, 13.3%, 40.0%, 44.4% and 15.6% in recipients respectively. While the allele frequencies of *ACE I/I*, *D/I*, and *D/D* were 44.4%, 41.1% and 14.5% in recipients respectively. The allele frequencies of *CYP3A5*, *ABCB1* at 1236C>T, 2677G>AT and 2677G>AT, and *ACE* were detailedly shown in Table 2.

**Table 1:** Demographic characteristics of recipients and donors.

	Recipient	Donor
Age (Median, Range)	10 (4–120 month)	30 (21–56 year)
Sex		
Male (%)	52 (57.8)	37 (41.1)
Female (%)	38 (42.2)	53 (58.9)
Body weight (Mean ± Sd; kg)	8.88±3.28	59.35±9.47
Height (Mean ± Sd; cm)	72.97±13.69	162±8.48
Surface area (m^2^)	0.41±0.12	
*CYP3A5* genotype		
AA, *1/*1 (%)	3 (3.3)	11 (12.2)
AG, *1/*3 (%)	37 (41.1)	34 (37.8)
GG, *3/*3 (%)	50 (55.6)	45 (50.0)
*ABCB1* genotype		
1236C>T CT (%)	38 (42.2)	
TT (%)	42 (46.7)	
CC (%)	10 (11.1)	
2677G>AT AT (%)	13 (14.4)	
GA (%)	11 (12.2)	
GG (%)	21 (23.4)	
GT (%)	33 (36.7)	
TT (%)	12 (13.3)	
3435C>T CC (%)	36 (40.0)	
CT (%)	40 (44.4)	
TT (%)	14 (15.6)	
*ACE* genotype		
* I/I* (%)	40 (44.4)	
* D/I* (%)	37 (41.1)	
* D/D* (%)	13 (14.5)	
Primary diseases		
Congenital biliary atresia (%)	89 (98.9)	
Postoperative chologenic infection (%)	1 (1.1)	

**Table 2:** Frequency of Genotyping from recipients and donors.

Genotype	Recipient	Donor	Tolerance/Total
CYP3A5	AA	AA	1/2
	AA	AG	0/1
	AA	GG	0/0
	AG	AA	2/8
	AG	AG	7/18
	AG	GG	5/11
	GG	AA	0/1
	GG	AG	7/15
	GG	GG	9/34
ABCB1 (1236C>T)	CC	CC	1/2
	CC	CT	3/8
	CC	TT	0/0
	CT	CC	2/7
	CT	CT	7/20
	CT	TT	3/11
	TT	CC	0/1
	TT	CT	8/19
	TT	TT	7/22
ABCB1 (2677G>AT)	AA	AA	0/0
	AA	AT	0/0
	AA	GA	0/0
	AA	GG	0/0
	AA	GT	0/0
	AA	TT	0/0
	AT	AA	1/2
	AT	AT	1/3
	AT	GA	2/4
	AT	GG	0/0
	AT	GT	1/2
	AT	TT	1/2
	GA	AA	0/0
	GA	AT	1/2
	GA	GA	1/1
	GA	GG	0/5
	GA	GT	0/3
	GA	TT	0/0
	GG	AA	0/0
	GG	AT	1/1
	GG	GA	2/5
	GG	GG	1/4
	GG	GT	3/9
	GG	TT	0/2
	GT	AA	0/0
	GT	AT	2/2
	GT	GA	0/2
	GT	GG	3/6
	GT	GT	5/15
	GT	TT	2/8
	TT	AA	0/0
	TT	AT	1/1
	TT	GA	0/0
	TT	GG	0/0
	TT	GT	2/5
	TT	TT	1/6
ABCB1 (3435C>T)	CC	CC	6/19
	CC	CT	3/17
	CC	TT	0/0
	CT	CC	3/11
	CT	CT	9/21
	CT	TT	3/8
	TT	CC	0/1
	TT	CT	5/7
	TT	TT	2/6
ACE	D/D	D/D	1/4
	D/D	D/I	1/9
	D/D	I/I	0/0
	D/I	D/D	2/9
	D/I	D/I	7/17
	D/I	I/I	4/11
	I/I	D/D	0/0
	I/I	D/I	3/8
	I/I	I/I	12/32

### 2. Effect of *CYP3A5* genotype in recipient (intestine) on TAC dosing requirements and disposition

According to *CYP3A5* genotypic results, pediatric recipients were divided into tow groups: expressor (*1/*1 and *1/*3 allele), and nonexpressor (*3/*3 allele). We compared clinical characteristics between two groups and showed them in Table 3. There was no significant difference in age, sex, body weight, height, primary diseases and postoperative complications between the recipients with expressor and those with nonexpressor. And there was no significant difference in donors’ age, sex, body weight and height between the recipients with expressor and those with nonexpressor.

**Table 3:** Comparison of characteristics of recipients by *CYP3A5* genotyping.

	Expressor	Nonexpressor	P value
Age (Mean ± Sd; month)	19.0±23.0	16.0±14.4	0.448
Sex			0.702
Male (%)	24 (60.0)	28 (56.0)	
Female (%)	16 (40.0)	22 (44.0)	
Body weight (Mean ± Sd; kg)	9.2±3.7	8.6±2.9	0.426
Height (Mean ± Sd; cm)	73.4±14.6	72.7±13.1	0.809
Surface area (m^2^)	0.41±0.13	0.40±0.12	0.663
*CYP3A5* genotype			
AA, *1/*1 (%)	3 (3.3)	0 (0)	
AG, *1/*3 (%)	37 (41.1)	0 (0)	
GG, *3/*3 (%)	0 (0)	50 (55.6)	
Primary diseases			0.368
Congenital biliary atresia (%)	40 (100.0)	49 (98.0)	
Postoperative chologenic infection (%)	0 (0)	1 (2.0)	
TAC peak time (day)	9.95±8.25	5.90±4.23	0.004
Donor			
Age (Mean ± Sd; year)	32.4±8.9	30.5+5.3	0.260
Male (%)	21 (52.5)	16 (32)	0.050
Female (%)	19 (47.5)	34 (68)	
Body weight (Mean ± Sd; kg)	59.2±9.9	59.5±9.2	0.891
Height (Mean ± Sd; cm)	164.4±8.0	164.2±8.9	0.929
AA, *1/*1 (%)	10 (25.0)	1 (2.0)	
AG, *1/*3 (%)	19 (47.5)	15 (30.0)	
GG, *3/*3 (%)	11 (27.5)	34 (68.0)	0.000

Note: Expressor, *CYP3A5* *1/*1 and *1/*3; Nonexpressor, *CYP3A5* *3/*3.

**Figure 1:**
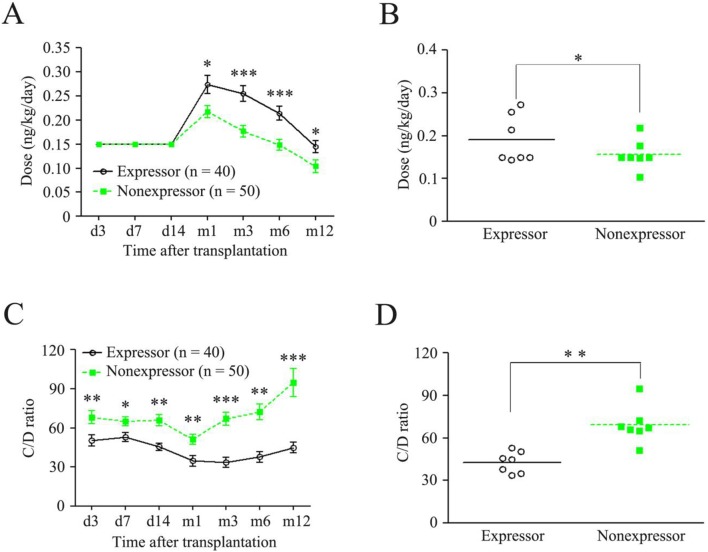
Doses and C/D ratios of TAC compared between recipients with *CYP3A5* expressor and those with nonexpressor. (A) Dose-time curves; (B) Doses in dots, every dot represents a dose at a time point; (C) C/D ratio-time curves; (D) C/D ratios in dots, every dot represents a C/D ratio at a time point. TAC, tacrolimus; Expressor, *CYP3A5* *1/*1 and *1/*3; Nonexpressor, *CYP*3A5 *3/*3; *p<0.05; **p<0.01; ***p<0.001.

However, the peak time of TAC in the recipients with expressor was significantly longer than that in the recipients with nonexpressor (9.95±8.25 *vs.* 5.90±4.23, p<0.01; **Table 3**). We further investigated the difference of dose and C/D ratio between the recipients with expressor and those with nonexpressor. As shown in Figure 1, although the two groups had the same TAC initial dose (ng/kg/day) and early induction dose (from day 3 to day 14, **Fig. 1A and 1B**), the C/D ratio in the recipients with nonexpressor was significantly higher than those with expressor (**Fig. 1C and 1D**). And then a higher TAC dose was adjusted on day 14 after transplantation according to their C/D ratio in both expressor and nonexpressor groups. Importantly, the highest dose used on day 30 was almost two-fold of the initial dose in the recipients with expressor, which was significantly higher than that in the recipients with nonexpressor at the same time point (0.27±0.12 *vs.* 0.22±0.09, p = 0.013; **Fig. 1A**). Then the TAC maintenance dose was progressively reduced in both groups. At the month12 time point, the TAC dose was almost reached the initial dose in the recipients with expressor (0.14±0.06), whereas the dose was lower than the initial dose in those with nonexpressor (0.10±0.06; **Fig. 1A**). While the normalized trough concentrations, the C/D ratios in the recipients with nonexpressor were significantly higher than those in the recipients with expressor at every time points during a year following transplantation (**Fig. 1C**). Therefore, the correlation between *CYP3A5* genotype and TAC late induction and maintenance doses was observed: expressor group had higher doses and lower C/D ratios, whereas nonexpressor group had lower doses and higher C/D ratios (**Fig. 1B and 1D**). Those results indicate that the recipients with expressor require more time to achieve TAC therapeutic range during the induction phase, need more upward dose during the late induction and the maintained phases, and have lower C/D ratio. In contrast, the recipients with nonexpressor require less time to achieve TAC therapeutic range during the induction phase, need lower dose during the late induction and the maintained phases, and have higher C/D ratio.

### 3. Impact of *CYP3A5* genotype in donors (graft liver) on TAC dosing requirements and disposition

**Figure 2:**
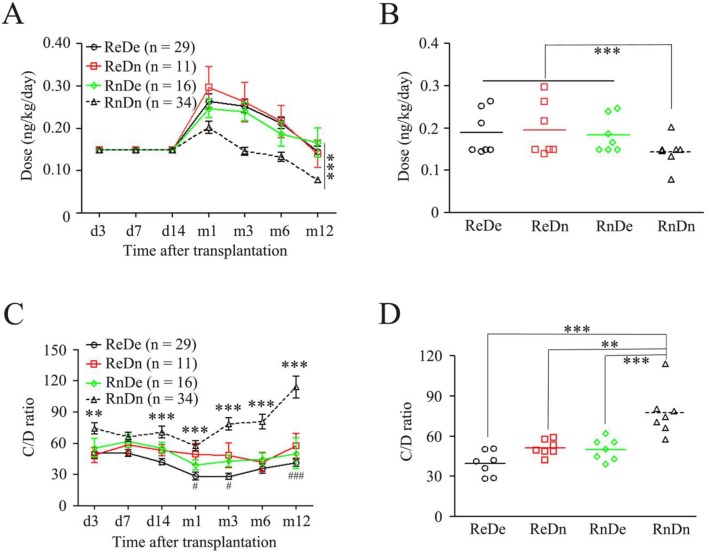
Doses and C/D ratios of TAC compared among four groups: ReDe, ReDn, RnDe and RnDn. (A) Dose-time curves; (B) Doses in dots, every dot represents a dose at a time point; (C) C/D ratio-time curves; * compared with ReDe; # compared with ReDn; (D) C/D ratios in dots, every dot represents a C/D ratio at a time point. TAC, tacrolimus; Expressor, *CYP*3A5 *1/*1 and *1/*3; Nonexpressor, *CY*P3A5 *3/*3; ReDe, recipient with expressor/donor with expressor; ReDn, recipient with expressor/donor with nonexpressor; RnDe, recipient with nonexpressor/donor with expressor; RnDn, recipient with nonexpressor/donor with nonexpressor; *p<0.05; **p<0.01; ***p<0.001; #p<0.05, ###p<0.001.

There was significant difference in donors’ *CYP3A5* genotypes between the recipients with expressor and those with nonexpressor, when Chi-square test was used (**Table 3**). We therefore further investigated whether *CYP3A5* expressor and nonexpressor from donors affect TAC dosing requirements and C/D ratio of recipients. According to recipients’ and donors’ *CYP3A5* genotyping, pediatric recipients were divided into four groups: the recipients with expressor/the donor with expressor (ReDe), the recipients with expressor/the donor with nonexpressor (ReDn), the recipients with nonexpressor/the donor with expressor (RnDe) and the recipients with nonexpressor/the donor with nonexpressor (RnDn). We found that the initial, induction and maintenance doses are very close to those in ReDe, ReDn and RnDe groups, respectively, while the late induction and the maintenance doses in RnDn group were significantly less than those in other three groups (**Fig. 2A and 2B**). However, TAC C/D ratios were observed with a different phenotype compared with TAC dosing phenotypes. With time, C/D ratio in RnDn group significantly increasingly higher than those in other three groups, especially in the maintenance phase (**Fig. 2C**). Moreover, ReDn group had higher C/D ratio than ReDe at month1, month3 and month12 time points (**Fig. 2C**). The overall dosing in very group was analyzed and showed in Figure 2D. The RnDn group had significantly higher TAC C/D ratio than other three groups. Although ReDn and RnDe groups had higher C/D ratio than ReDe group, no statistically significant relationship was observed among them (**Fig. 2D**). More importantly, the RnDe group had significantly lower C/D ratio than RnDn group (**Fig. 2D**), suggesting that although two groups share the same intestine *CYP3A5* expressor, graft livers with *CYP3A5* expressor or with *CYP3A5* nonexpressor play important impact on TAC trough concentrations after liver transplantation.

### 4. Effects of *ABCB1* and *ACE* genotypes in recipient (intestine) on TAC dosing requirements and disposition

**Figure 3:**
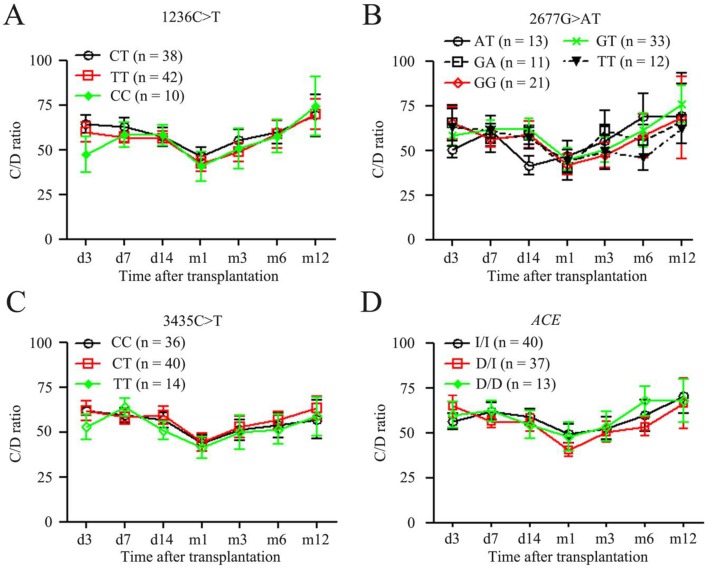
C/D ratios of TAC compared among *ABCB1* genotypes and among *ACE* genotypes. (A) C/D ratio-time curves of *ABCB1* variants at 1236C>T; (B) C/D ratio-time curves of *ABCB1* variants at 2677G>AT; (C) C/D ratio-time curves of *ABCB1* variants at 3435C>T; (D) C/D ratio-time curves of *ACE* variants. TAC, tacrolimus.

Considering the possible influence of *ABCB1* and *ACE* SNPs in recipients on TAC pharmacokinetics, we finally assessed the effects of SNPs of *ABCB1* and *ACE* in intestine on TAC. As shown in Figure 3, we didn’t find any significant difference of the C/D ratios among the recipients with *ABCB1* at position 1236CT, TT and CC (**Fig. 3A**), among those with *ABCB1* at position 2677AT, GT, GG, GT and TT (**Fig. 3B**), among those with ABCB1 at position 3435CC, CT and TT (**Fig. 3C**), and among those with *ACE* at intron 16 (14901–14378) (**Fig. 3D**). These results indicate that the variants of *ABCB1* and *ACE* have minimal impact on TAC disposition in pediatric liver transplant patients.

### 5. Analysis of relationship between donors and recipients

We investigated the family relationship between donors and recipients. As shown in Table 4, parental relationship between donors and recipients with CYP3A5*1/*1, *CYP3A*5*1/*3 and CYP3A5*3/*3 were 2 (2.2%), 35 (38.9%) and 48 (53.3%) cases respectively, whereas grandparental relationship between those were 1 (1.1%), 2 (2.2%) and 2 (2.2%) cases respectively. Parental relationship between those with *ABCB1* 1236CT, TT, CC, 2677AT, GA, GG, GT, TT, 3435CC, CT and TT were 37 (41.1%), 39 (43.3%), 9 (10.0%), 12 (13.3%), 11 (12.2%), 20 (22.2%), 31 (34.4%), 11 (12.2%), 34 (37.8%), 38 (42.2%) and 13 (14.4%) respectively, whereas grandparental relationship between those were 1 (1.1%), 3 (3.3%), 1 (1.1%), 1 (1.1%), 0 (0%), 1 (1.1%), 2 (2.2%), 1 (1.1%), 2 (2.2%), 2 (2.2%) and 1 (1.1%) cases respectively. Parental relationship between those with *ACE I/I*, *D/I*, and *D/D* were 38 (42.2%), 36 (40.0%) and 11 (12.2%) cases respectively, whereas grandparental relationship between those were 2 (2.2%), 1 (1.1%) and 2 (2.2%) cases respectively.

**Table 4:** Relationship between recipients and donors.

Genotype of recipients	Recipients’ relationship to donors	
	Parents (%)	Grandparents (%)
*CYP3A5* genotype		
AA, *1/*1	2 (2.2)	1 (1.1)
AG, *1/*3	35 (38.9)	2 (2.2)
GG, *3/*3	48 (53.3)	2 (2.2)
*ABCB1* genotype		
1236C>T CT	37 (41.1)	1 (1.1)
TT	39 (43.3)	3 (3.3)
CC	9 (10.0)	1 (1.1)
2677G>AT AT	12 (13.3)	1 (1.1)
GA	11 (12.2)	0 (0)
GG	20 (22.2)	1 (1.1)
GT	31 (34.4)	2 (2.2)
TT	11 (12.2)	1 (1.1)
3435C>T CC	34 (37.8)	2 (2.2)
CT	38 (42.2)	2 (2.2)
TT	13 (14.4)	1 (1.1)
*ACE* genotype		
* I/I*	38 (42.2)	2 (2.2)
* D/I*	36 (40.0)	1 (1.1)
* D/D*	11 (12.2)	2 (2.2)

We further analyzed frequency and rejection of pairing genotype of donors and recipients. As shown in Table 5, recipients with concomitant rejection in the same and different genotyping of donors with recipients in *CYP3A5* were 15 (34.1%) and 11 (30.6%) cases respectively. Recipients with concomitant rejection in the same and different genotyping of donors and recipients in *ABCB1* at 1236, 2677 and 3435 sites were 15 (34.1%), 11 (23.9%), 6 (20.7%), 21 (34.4%), 14 (30.4%) and 12 (27.3%) respectively. While recipients with concomitant tolerance in the same and different genotyping of donors and recipients in *ACE* were 12 (22.6%) and 14 (37.8%) respectively. Interestingly, no statistical difference between those with and without concomitant rejection in the same and different genotyping, including *CYP3A5*, *ABCB1* and *ACE*, was observed.

**Table 5:** Profiles of pairing genotypes of donors and recipients on Rejection of TAC.

Genes		Donor and recipient	Rejection (total)	Non-rejection (total)	P value
CYP3A5		Same genotype	15 (54)	39 (54)	
		Different genotypes	11 (36)	25 (36)	0.776
ABCB1					
	1236C>T	Same genotype	15 (44)	29 (44)	
		Different genotypes	11 (46)	35 (46)	0.175
	2677G>AT	Same genotype	6 (29)	23 (29)	
		Different genotypes	21 (61)	40 (61)	0.170
	3435G>AT	Same genotype	14 (46)	32 (46)	
		Different genotypes	12 (44)	32 (44)	0.110
ACE		Same genotype	12 (53)	41 (53)	
		Different genotypes	14 (37)	23 (37)	0.105

## Discussion

Therapeutic drug monitoring of TAC in blood is necessary to provide an effective immunosuppression and avoid adverse effects after organ transplantation. With regard to TAC pharmacokinetic variability, *CYP3A5* genotype has been reported to consistently associate with TAC dosing requirement [9]. In pediatric recipients, however, it is difficult to perform frequently blood samplings for measurement. Therefore, it is very important to investigate the relationship of *CYP3A5* genotyping with TAC pharmacokinetics for establishing a personalized dosage regimen including the initial, the induction and the maintenance doses. In this study, the general consistency in the concept that *CYP3A5* expressor requires higher TAC doses than nonexpressor to reach target trough concentrations strongly suggests that *CYP3A5* genotyping not only in recipient (intestine) but also in donor (graft liver) is necessary for establishing a personalized dosage regimen in pediatric liver transplant patients. In addition, we didn’t find any significant impact of *ABCB1* and *ACE* SNPs on TAC disposition. Although a recent study suggested a safer dosing and monitoring of TAC coadministered with rabeprazole early on after liver transplantation regardless of *CYP3A5* genotypes of recipients and their donors [18], our finding in this study is important as it emphasizes the combined effects of recipient’s and donor’s genetic variation in relation to TAC disposition. Moreover, although primary outcome time focused on the early postoperative period in most studies, we set one year of primary outcome time. It is necessary, we think, because impact on recipients, especially for pediatric liver transplant patients, will be long time period because of immunosuppressive regimen for his whole life.

TAC is characterized by narrow therapeutic index and interindividual variability in its exposure, and achieving target therapeutic level is difficult, especially during the early period of transplantation. Therefore, the TAC dosing regimens require a regular drug monitoring system based on its trough blood concentration [9]. On the other hand, TAC blood concentration is monitored to allow therapeutic levels to be maintained, to avoid toxicity and to improve efficacy. In general, post-operative infections were considered as over-immunosupressive, which needs to reduce the dose of TAC, whereas acute cellular rejection was considered as under-immunosupressive, which needs to increase the dose of TAC. But the former needs to exclude the ordinary post-operative infections. Although the same initial TAC dose and the same early induction dose (∼two weeks) were used, we were surprised to find a *CYP3A5* genotype effect so early after transplant, in which the C/D ratio was significantly higher in nonexpressor than expressor on day 3 (**Fig. 1**). For pediatric liver recipients, in contrast, another study claimed that they did not identify any relationship between recipient *CYP3A5* genotype and TAC dosing [19]. They supposed that the main reason for this lack of association was probably that variations in TAC deposit are largely dependent on hepatic metabolism and to a lesser extent on intestinal metabolism in the first 14 days after transplantation [19]. In addition, a similar data in pediatric renal transplant recipients have also shown that the independent impact of *CYP3A5* genotype on TAC pharmacogenetic was not evident [20]. We postulate that the main reason for this inconsistence with our results is probably that we had enrolled more cases, demonstrating a further large-scale study is necessary. Although CYP3A5 genotype has been convincingly impacted on TAC clearance in many ethic groups [1]–[3], [5]–[7], [9]–[12], [16], [17], [19], [21], [22], there is limited evidence to prove that *CYP3A5* genotype-guided TAC dosing will benefit clinical outcomes.

In this study, we found that the association between TAC dosing and *CYP3A5* genotyping not only in recipients but also in donors for liver transplantation (**Fig. 1** and **Fig. 2**). Recent a report has also shown that a more significant effect of donor genotype as early as 2 weeks after transplantation in liver transplant recipients [23]. In any case, the relative importance of recipient and donor genotyping during the early post-transplantation period is of particular significance, especially in liver transplantation, concerning the risk of graft rejection is the highest in this period. To maximize the immunosuppressive effect and minimize adverse effects, TAC dosing regimen of in the induction phase (∼3 months after transplantation) and the maintenance phase (3–12 months after transplantation) should be changed [7]. Generally, TAC dosing requirement for the induction phase is higher than that requirement for the maintenance phase. In the present study, a higher TAC dose was adjusted on day 14 after transplantation basing on C/D ratio monitor. The highest dose used on day 30 was almost two-fold of the initial dose in the recipients (**Fig. 1A**), then the TAC maintenance dose was progressively reduced, and on day 365 after transplantation, the TAC dose was almost reduced to the initial dose in the recipients (**Fig. 1A**). It is very clear that pharmacogenetics-based approach to TAC dosing may prove to be more clinically relevant in terms of preventing early overexposure and toxicity. Possibly, starting with a lower TAC dose in such patients may prevent early nephrotoxicity or the development of new-onset diabetes after transplantation.

Patients with *CYP3A5* expressor require a higher TAC dose than *CYP3A5* nonexpressers to reach the same whole-blood exposure. Therefore, these expressor patients are prone to have subtherapeutic drug concentrations in the early phase after surgery and theoretically maybe increase acute rejection risk [9]. It is not surprising that, from a clinical point of view, TAC is prescribed to prevent acute rejection. However, an exception to this general pattern is a study of Korean kidney graft patients, which found a greater incidence of acute rejection with *CYP3A5* expressor [11]. A previous study reported that children younger than five years of age needed higher TAC doses than older children after both kidney and liver transplant and suggested that TAC starting dosing guidelines in children should reflect both age and *CYP3A5* genotype to quickly reach therapeutic concentrations after transplantation [19]. However, we didn’t find an association between age and *CYP3A5* genotype (Table 3). We supposed that pediatric recipients in our study caused this inconsistence, which are almost younger children with only few cases above 5 years of age. Regarding that our results revealed the influence of CYP3A5 variant recipient or donor genotypes on TAC metabolic variables, we did not agree the idea that the impact of age and genetic variation appears to be weakened in the immediate post-transplantation period, while intraindividual variation appears larger [21].

The wide range of interpatient variability in effective dosage of TAC is caused by various factors, such as age, weight, and drug interactions. Similarly, inflammation and/or organ failure maybe reduce drug metabolism in patients [19]. In particular, genetic factors play an important role in the pharmacokinetic properties and therapeutic levels of TAC. The *CYP3A5* genotype is currently the strongest predictor of an individual’s TAC dose requirement. However, it does not explain all variability. Other genetic variants may explain additional variation in TAC dose requirement. As has been illustrated in adults, the drug transporter ABCB1, the CYP3A4, the human pregnane X receptor (NR1I2), interleukin 6 and COMT SNP may be associated with early TAC exposure [9], [24]–[26]. Although the association of *ABCB1*, *ACE* with the C/D ratios of TAC was investigated, we didn’t observe any significant impact on TAC disposition in pediatric liver transplants (**Fig. 3**). Although a few reports found that high intestinal levels of P-glycoprotein were associated with TAC disposition after liver transplantation [16], [27], most studies didn’t find any influence of ABCB1 genotypes on TAC pharmacokinetics [28]–[30], especially in pediatric recipients [6], [31], [32]. Consistent with the majority, we couldn’t find any significant association between ABCB1 genotypes and the disposition of TAC (**Fig. 3**). With regard to *ACE* SNPs, although the *ACE* study suggested an association between *ACE* and renal dysfunction in adult liver recipients who receive TAC [32], we couldn’t find any significant association between its genotypes and the disposition of TAC too (**Fig. 3**). Taken together, their influence appears to be smaller than that of *CYP3A5* SNPs. If these additional genetic variants do indeed explain residual variability in TAC dose requirement, it may become possible to develop personalized therapeutic strategy that helps clinicians to decide on an individual’s initial dose. It is to be expected that with such an approach early TAC overexposure and toxicity may be expectantly prevented [9]. Further prospective studies of liver transplant recipients are needed to evaluate the impact of these genetic polymorphisms on TAC dosing requirement and determine whether routine genotyping would be improve in personalized TAC therapy. Since the actions of these genes appear to be cooperative. Moreover, the combination of some drugs with lower TAC dose may be safely coadministered [33]. However, our results provided evidence that *CYP3A5* plays a more dominant role than other genetic variants in the metabolism of TAC in pediatric liver transplant recipients and their donors.

In this study, we analyzed the relationship of pairing of donors and recipients. Among pairing of donors and recipients, parental relationship cases were more than grandparental those (Table 4). In addition, there were not significantly different in occurrence of rejection between the same or different genotypes in pairing of donors and recipients (Table 5). In additional, not only donors but also recipients were genotyped with their peripheral blood samples. It seems no difference for genotyping regardless of basing on intestinal biopsies or blood samples, but using intestinal biopsies will have high novelty, especially for recipients. More importantly, intestinal biopsies from recipients will provide us more valued information about mRNA transcription and protein expression of interesting genes and second pass of metabolism of TAC.

The main limitations of this study are the retrospective design from a single center and a limited number of patients. Also, the confounding effects of *CYP3A4* with *ABCB1* or *ACE* variants that may affect TAC pharmacokinetics were not examined. A prospective study with a large number of pediatric recipients and standard timing of ImmuKnow assay is required to establish an effective monitoring tool of immune response in children following liver transplantation. Furthermore, for recipient genotyping, periphery blood has limited novelty.

In conclusion, this study further confirmed that the *CYP3A5* polymorphism at position 6986G>A of pediatric liver transplants and their donors, but not *ABCB1* or *ACE* SNPs in recipients, impacts on TAC dosing requirement, suggesting that early determination of the *CYP3A5* genotype in both recipients and donors would be helpful in the design of adequate immunosuppressive treatment and in lower adverse effects by predicting TAC dosing requirement for the induction and maintenance phases in individual liver transplant recipients.
